# Short- and Long-Term Biomarkers for Bacterial Robustness: A Framework for Quantifying Correlations between Cellular Indicators and Adaptive Behavior

**DOI:** 10.1371/journal.pone.0013746

**Published:** 2010-10-29

**Authors:** Heidy M. W. den Besten, Aarathi Arvind, Heidi M. S. Gaballo, Roy Moezelaar, Marcel H. Zwietering, Tjakko Abee

**Affiliations:** 1 Top Institute Food and Nutrition, Wageningen, The Netherlands; 2 Laboratory of Food Microbiology, Wageningen University and Research Centre, Wageningen, The Netherlands; 3 Food and Biobased Research, Wageningen University and Research Centre, Wageningen, The Netherlands; Fundació Institut Germans Trias i Pujol, Universitat Autònoma de Barcelona, CibeRES, Corporate Research Program on Tuberculosis (CRP-TB), Spain

## Abstract

The ability of microorganisms to adapt to changing environments challenges the prediction of their history-dependent behavior. Cellular biomarkers that are quantitatively correlated to stress adaptive behavior will facilitate our ability to predict the impact of these adaptive traits. Here, we present a framework for identifying cellular biomarkers for mild stress induced enhanced microbial robustness towards lethal stresses. Several candidate-biomarkers were selected by comparing the genome-wide transcriptome profiles of our model-organism *Bacillus cereus* upon exposure to four mild stress conditions (mild heat, acid, salt and oxidative stress). These candidate-biomarkers—a transcriptional regulator (activating general stress responses), enzymes (removing reactive oxygen species), and chaperones and proteases (maintaining protein quality)—were quantitatively determined at transcript, protein and/or activity level upon exposure to mild heat, acid, salt and oxidative stress for various time intervals. Both unstressed and mild stress treated cells were also exposed to lethal stress conditions (severe heat, acid and oxidative stress) to quantify the robustness advantage provided by mild stress pretreatment. To evaluate whether the candidate-biomarkers could predict the robustness enhancement towards lethal stress elicited by mild stress pretreatment, the biomarker responses upon mild stress treatment were correlated to mild stress induced robustness towards lethal stress. Both short- and long-term biomarkers could be identified of which their induction levels were correlated to mild stress induced enhanced robustness towards lethal heat, acid and/or oxidative stress, respectively, and are therefore predictive cellular indicators for mild stress induced enhanced robustness. The identified biomarkers are among the most consistently induced cellular components in stress responses and ubiquitous in biology, supporting extrapolation to other microorganisms than *B. cereus*. Our quantitative, systematic approach provides a framework to search for these biomarkers and to evaluate their predictive quality in order to select promising biomarkers that can serve to early detect and predict adaptive traits.

## Introduction

Bacteria are constantly faced with changing environmental conditions and have evolved sophisticated mechanisms to adapt to changing environments. Environmental changes trigger a cascade of cellular events, and regulatory networks of microorganisms serve to prime cells to be prepared for later challenges even before they arise [1], [2]. The adaptive stress response is a crucial survival strategy for a wide spectrum of microorganisms, including food spoilage bacteria, pathogens and organisms used in functional food applications, and prediction of this stress adaptive behavior will allow to control and/or exploit these adaptive traits.

Bacteria are not only exposed to changing environments in their natural habitats but also during industrial processing, and activation of stress adaptation mechanisms can provide cell robustness to harsher stress conditions including stresses other than the one that induced the adaptive stress response. This so-called cross-protection phenomenon challenges the hurdle preservation strategy that targets to guarantee the microbial safety and stability as well as the sensory and nutritional quality of minimally processed foods by simultaneous or successive application of multiple mild preservation factors [3]. While individual hurdles may not be effective in controlling growth of food spoilage bacteria and pathogens, the right combination of hurdles allows to control growth of microorganisms and to minimize organoleptic changes in foods. However, the ability of spoilage and pathogenic microorganisms to adapt to stressing environments could antagonize the benefits of the hurdle preservation strategy. Long- and short-term exposure to mild stress conditions showed to induce (cross-)protection towards otherwise lethal stress conditions [4], [5], [6] and might even affect the virulence of pathogens [7], [8]. The ability of microorganisms to gain cellular robustness upon activation of adaptive stress responses is also beneficial for various industrial applications [9], including the development of reliable starter cultures [10] and selection of robust probiotic strains [11].

Previous studies demonstrated that our model-organism *Bacillus cereus* gained increased resistance upon preexposure to several mild food preservation stresses [12]–[15], underlining the significance of a better understanding of its stress adaptive behavior. The availability of complete genome sequences of microbes and the development of functional genomics technologies have provided a wealth of data and opportunities to better understand stress adaptation mechanisms. Comparison of genome-wide transcriptome profiles of microorganisms in response to diverse environmental conditions can reveal general stress response features [16]–[19], and could lead to identification of cellular indicators for stress adaptive behavior. Recently, we investigated the genome-wide transcriptome response of *B. cereus* during exposure to several mild stresses [20]–[23], and these transcriptome analyses gave insight into general and stress-specific adaptive responses, and opened avenues towards identification of potential cellular biomarkers that can predict stress adaptive behavior. Here, we present a framework for the identification of such cellular biomarkers for mild stress induced enhanced robustness towards lethal stresses. We identified both short- and long-term biomarkers of which their induction levels were quantitatively correlated to the mild stress induced enhanced robustness level of the cell. The identification of predictive cellular indicators for stress adaptive behavior will enable the prediction of the impact of adaptive responses during mild stress (processing) conditions on subsequent microbial robustness, and can be exploited to control these stress adaptive traits.

## Results

### Experimental strategy for identifying biomarkers for mild stress induced enhanced robustness

**Figure 1:**
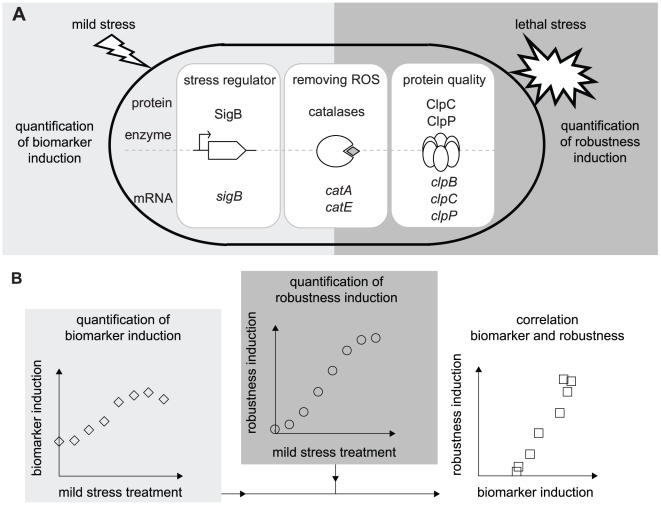
Conceptual scheme for measuring and correlating mild stress induced biomarker and robustness responses. a) Candidate-biomarkers - a transcriptional regulator (activating general stress responses), catalases (removing reactive oxygen species, ROS), and chaperones and proteases (maintenance of protein quality) - were quantitatively measured at transcript, protein and/or activity level upon exposure to four mild stress conditions (43°C, pH 5.5, 1.5% NaCl, 0.1 mM H_2_O_2_) for various time intervals. Mild stress treated cells were also exposed to three lethal stresses (50°C, pH 3.3, 0.2 mM H_2_O_2_) to quantify their robustness towards these lethal stresses following mild stress pretreatment. b) To evaluate whether the candidate-biomarker could predict the robustness level of mild stress pretreated cells, the mild stress induced biomarker and robustness responses were correlated to evaluate the significance of their correlation.

In search of potential biomarkers for mild stress induced enhanced robustness, the genome-wide transcriptome profiles of our model-organism *B. cereus* ATCC 14579 grown until the mid-exponential growth phase and then exposed to various mild stress conditions [20]–[23] were compared. This comparison revealed a remarkable limited number of genes (see Table S1 in the supplementary material) that were differentially expressed upon treatment to all those mild stress conditions. This overlap of transcriptome responses included various defense mechanisms and regulatory and metabolic pathways, including cellular defense mechanisms against oxidative stress, factors involved in repair and maintenance of cellular protein quality and energy production, and transcriptional regulators. In order to select the most promising functional categories that could point to candidate-biomarkers, the expression ratios of the differentially expressed genes were evaluated by marking the genes of which the expression ratios were at least five upon exposure to one mild stress condition and at least two upon exposure to two other mild stress conditions. These genes represented three main functional categories: (1) members of the general stress response regulon controlled by transcriptional regulator σ^B^; (2) cellular defense mechanisms against oxidative stress; and (3) repair and maintenance of cellular protein quality. This transcriptional overlap in stress adaptation seemed to be stress-independent, supporting its suitability as a source for identification of potential candidate-biomarkers for stress adaptation. Based on these three categories, candidate-biomarker transcripts for stress adaptive behavior were assigned, namely that of *sigB*, *catA*, *catE*, *clpB*, *clpC* and *clpP*. Conceivably, not only transcripts but also proteins and enzymes could function as biomarker, and therefore, also the proteins SigB, ClpC and ClpP, and catalase activity were included as candidate-biomarkers, resulting in ten candidate-biomarkers (Figure 1a).

To quantify the stress adaptive behavior of *B. cereus* to a wide spectrum of stress conditions imposed on bacteria, four mild stress conditions were used to mildly stress the cells for various time intervals, namely, heat stress (43°C), acid-shock (pH 5.5), osmotic-upshift (1.5% NaCl), and oxidative stress (0.1 mM H_2_O_2_) (see Figure S1 in the supplementary material for more details about the procedure to select these mild stress conditions for adaptation experiments). Both unstressed and mild stress treated cells were subsequently exposed to three lethal stress inactivation conditions − heat stress (50°C), low pH shock (pH 3.3) and high oxidative stress (0.2 mM H_2_O_2_) to determine their specific robustness level towards these lethal stresses. Severe osmotic-upshift (up to 30% sodium chloride [w/v]; reaches maximum saturation of sodium chloride in BHI) did not result in inactivation patterns comparable to those observed with the other three lethal stress conditions (data not shown) and was therefore not used as lethal stress. To quantify the robustness enhancement towards these lethal stresses provided by mild stress pretreatment, the robustness level of mild stress pretreated cells was compared to that of unstressed cells. To evaluate whether one or more of the selected candidate-biomarkers could quantitatively predict the robustness enhancement following mild stress pretreatment, the induction of the candidate-biomarkers was determined upon mild stress treatment and correlated to mild stress induced enhanced robustness towards lethal heat, acid and oxidative stress (Figure 1b). After correlating the mild stress induced biomarker and robustness responses, the Pearson correlation coefficient was calculated to evaluate the significance of the correlations in order to select potential biomarkers for mild stress induced enhanced robustness (Figure 1b).

### Induction of candidate-biomarkers and robustness towards lethal stress in response to mild stress treatment

**Figure 2:**
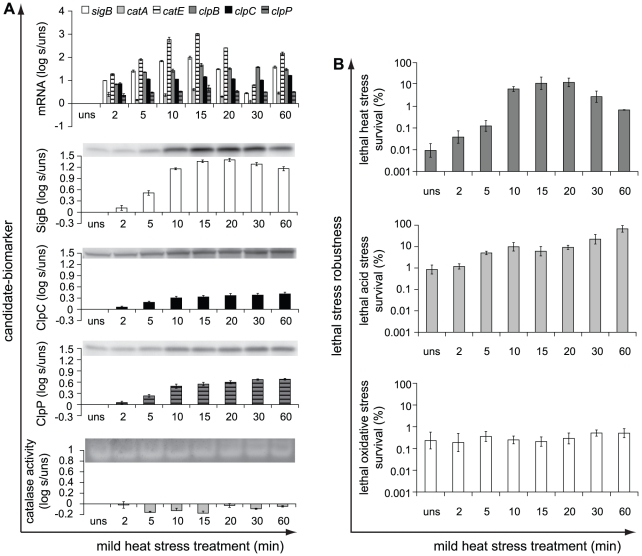
Induction of candidate-biomarkers and robustness in response to mild heat stress treatment. a) Candidate-biomarkers − the transcripts of *sigB*, *catA*, *catE*, *clpB*, *clpC* and *clpP*, the proteins SigB, ClpC and ClpP, and catalase activity − were quantitatively measured in unstressed (uns) cultures (OD_600 nm_ ∼0.4–0.5) and in the stressed cultures (s) after 43°C stress treatment for 2, 5, 10, 15, 20, 30 and 60 min. The columns mark the log_10_-fold induction after mild heat stress treatment compared to unstressed cells. b) Unstressed and 43°C-treated cells were inactivated by exposure to 50°C for 5 min, pH 3.3 at 30°C for 3.5 min, and 0.2 mM H_2_O_2_ at 30°C for 2 min, to determine their robustness towards lethal heat, acid and oxidative stress, respectively. The columns mark the number of microorganisms surviving the lethal stress treatment compared to the initial number of microorganisms (%). Error bars represent standard errors of the reproductions.

Mild heat stress treatment resulted in induction of almost all candidate-biomarkers, but their induction patterns differed (Figure 2a). The relative transcription levels of *sigB* and *catE*, which is a regulon member of σ^B^ [23], [24], displayed transient increased expression up to 15 min of mild heat stress treatment and decreased afterwards, whereas the transcription patterns of *clpB*, *clpC* and *clpP* were rather constant over time. Upon 60 min of mild heat stress treatment, the relative transcription levels of *sigB* and *catE* increased again, and this is in correspondence with upregulation of SigB-dependent genes in *B. subtilis* cells entering the transition growth phase [25]. The gene encoding the main vegetative catalase, *catA*, was just significantly transcribed at one mild heat stress treatment time point, and also, no increased catalase activity was observed in mild heat stress treated cells compared to unstressed cells. This confirmed that the role of catalase CatE is less significant in total cellular catalase activity compared to the main vegetative catalase CatA [26]. Mild heat stress treatment resulted in increased production of the proteins SigB, ClpC and ClpP compared to that of unstressed cells and the production patterns of both Clp proteins were comparable.

Mild heat stress pretreatment provided a robustness advantage towards lethal heat stress treatment, and also conferred cross-protection towards lethal acid stress, whereas no increased resistance towards lethal oxidative stress was observed upon mild heat stress pretreatment (Figure 2b).

**Figure 3:**
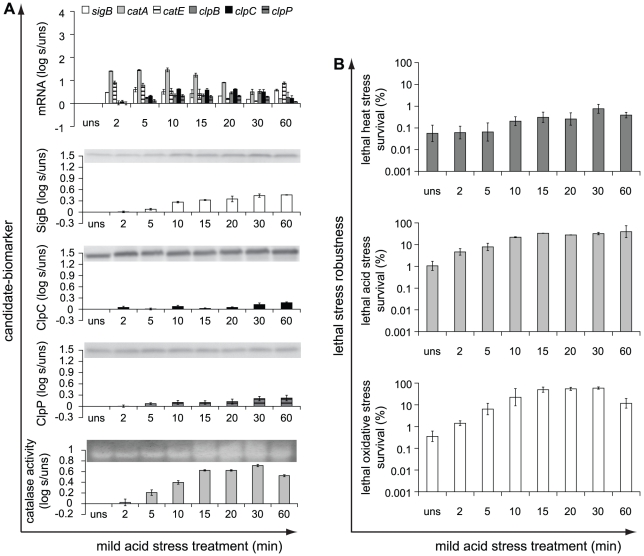
Induction of candidate-biomarkers and robustness in response to mild acid stress treatment. a) Candidate-biomarkers − the transcripts of *sigB*, *catA*, *catE*, *clpB*, *clpC* and *clpP*, the proteins SigB, ClpC and ClpP, and catalase activity − were quantitatively measured in unstressed (uns) cultures (OD_600 nm_ ∼0.4–0.5) and in the stressed cultures (s) after pH 5.5 stress treatment for 2, 5, 10, 15, 20, 30 and 60 min. The columns mark the log_10_-fold induction after mild acid stress treatment compared to unstressed cells. b) Unstressed and pH 5.5-treated cells were inactivated by exposure to 50°C for 5 min, pH 3.3 at 30°C for 3.5 min, and 0.2 mM H_2_O_2_ at 30°C for 2 min, to determine their robustness towards lethal heat, acid and oxidative stress, respectively. The columns mark the number of microorganisms surviving the lethal stress treatment compared to the initial number of microorganisms (%). Error bars represent standard errors of the reproductions.

**Figure 4:**
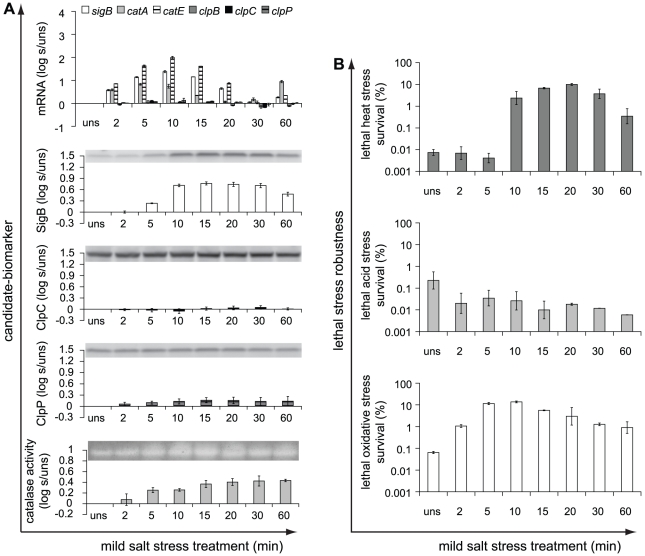
Induction of candidate-biomarkers and robustness in response to mild salt stress treatment. a) Candidate-biomarkers − the transcripts of *sigB*, *catA*, *catE*, *clpB*, *clpC* and *clpP*, the proteins SigB, ClpC and ClpP, and catalase activity − were quantitatively measured in unstressed (uns) cultures (OD_600 nm_ ∼0.4–0.5) and in the stressed cultures (s) after 1.5% NaCl stress treatment for 2, 5, 10, 15, 20, 30 and 60 min. The columns mark the log_10_-fold induction after mild salt stress treatment compared to unstressed cells. b) Unstressed and 1.5% NaCl-treated cells were inactivated by exposure to 50°C for 5 min, pH 3.3 at 30°C for 3.5 min, and 0.2 mM H_2_O_2_ at 30°C for 2 min, to determine their robustness towards lethal heat, acid and oxidative stress, respectively. The columns mark the number of microorganisms surviving the lethal stress treatment compared to the initial number of microorganisms (%). Error bars represent standard errors of the reproductions.

**Figure 5:**
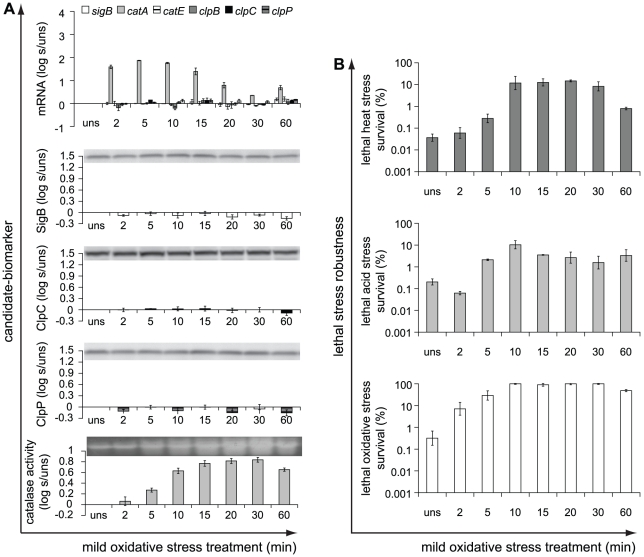
Induction of candidate-biomarkers and robustness in response to mild oxidative stress treatment. a) Candidate-biomarkers − the transcripts of *sigB*, *catA*, *catE*, *clpB*, *clpC* and *clpP*, the proteins SigB, ClpC and ClpP, and catalase activity − were quantitatively measured in unstressed (uns) cultures (OD_600 nm_ ∼0.4–0.5) and in the stressed cultures (s) after 0.1 mM H_2_O_2_ stress treatment for 2, 5, 10, 15, 20, 30 and 60 min. The columns mark the log_10_-fold induction after mild oxidative stress treatment compared to unstressed cells. b) Unstressed and 0.1 mM H_2_O_2_-treated cells were inactivated by exposure to 50°C for 5 min, pH 3.3 at 30°C for 3.5 min, and 0.2 mM H_2_O_2_ at 30°C for 2 min, to determine their robustness towards lethal heat, acid and oxidative stress, respectively. The columns mark the number of microorganisms surviving the lethal stress treatment compared to the initial number of microorganisms (%). Error bars represent standard errors of the reproductions.

Besides mild heat stress treatment, cells were also short- and long-term exposed to mild acid, salt and oxidative stress and the induction patterns of the candidate-biomarkers upon these mild stress treatments are shown in Figure 3a, 4a and 5a, respectively. The induction patterns of the candidate-biomarkers were highly affected by the type of mild stress condition applied. In addition to mild heat stress treatment, also mild acid and salt stress treatment significantly increased transcription of *sigB* and *catE* and led to increased SigB protein levels compared to unstressed cells, whereas these responses were not significant in mild oxidative stress treated cells. Mild acid stress treatment for up to 30 min elicited just significantly increased transcription of *clpB*, *clpC*, and *clpP* compared to unstressed cells, but increased production of the ClpC and ClpP proteins could not be demonstrated. Both mild salt and oxidative stress did not significantly increase the transcription of *clpB*, *clpC*, and *clpP* nor did these mild stress conditions lead to higher levels of the ClpC and ClpP proteins. In contrast to mild heat stress treatment, exposure to mild acid, salt and oxidative stress resulted in significantly increased transcription of *catA* and cellular catalase activity.

Mild acid stress pretreatment resulted in enhanced robustness towards lethal acid stress and provided also cross-protection towards lethal oxidative stress, whereas no significantly increased resistance towards lethal heat stress was observed (Figure 3b). On the other hand, mild salt stress pretreatment provided cross-protection towards lethal heat and oxidative stress, but did not confer cross-protection towards lethal acid stress (Figure 4b). Mild oxidative stress pretreatment provided cells enhanced robustness towards lethal heat, acid, and also oxidative stress, and therefore, only this mild stress condition conferred enhanced robustness to all three lethal stresses tested (Figure 5b). In Table S2 in the supplementary material, the effects of the different mild stress pretreatments on robustness towards lethal heat, acid and oxidative stress are summarized.

### Framework for identifying biomarkers for mild stress induced enhanced robustness

**Figure 6:**
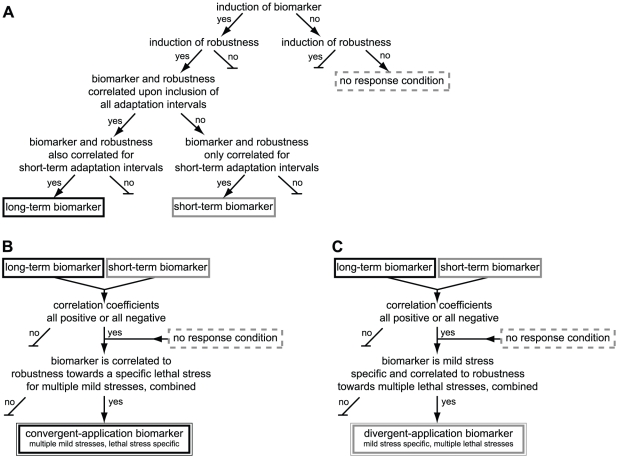
Framework for identification of biomarkers for mild stress induced enhanced robustness. a) Decision flow chart for identification of long-term and short-term biomarkers for mild stress induced enhanced robustness. Black box represents a long-term biomarker and gray box represents a short-term biomarker. These biomarkers are mild stress and lethal stress specific. Dashed gray box represents a no response condition. b) Decision flow chart for identification of a convergent-application biomarker that is induced by multiple mild stress conditions and is correlated to a specific lethal stress robustness. c) Decision flow chart for identification of a divergent-application biomarker that is induced by a specific mild stress condition and is correlated to robustness towards multiple lethal stresses.

A decision flow chart was designed to evaluate for each candidate-biomarker whether it could indeed predict the enhanced robustness level of mild stress pretreated cells, and therefore functioned as biomarker (Figure 6a). Two types of biomarkers were formulated, namely, long-term biomarkers and short-term biomarkers, and these two types of biomarkers will be discussed below. A step-wise procedure was followed to select those conditions where the candidate-biomarker functioned as biomarker. Firstly, the mild stress induced biomarker and robustness responses upon mild stress treatment for 2, 5, 10, 15, 20, 30 to 60 min (7 treatment time points) compared to that of unstressed cells were correlated for each mild stress and lethal stress pair, and the Pearson correlation coefficient was tested for significance (*P*<0.05). After testing this correlation, the treatment time point of 60 min was excluded from the analysis, after which the correlation of the remaining treatment time points was tested for significance. The exclusion procedure was followed until the exclusion of treatment time point of 10 min. When the mild stress induced candidate-biomarker and robustness responses were significantly correlated for all mild stress treatment time intervals, then the candidate-biomarker was qualified as long-term biomarker for that mild stress and lethal stress pair. The candidate-biomarker was qualified as short-term biomarker when the mild stress induced biomarker and robustness responses were only significantly correlated for short-term adaptation time intervals.

**Figure 7:**
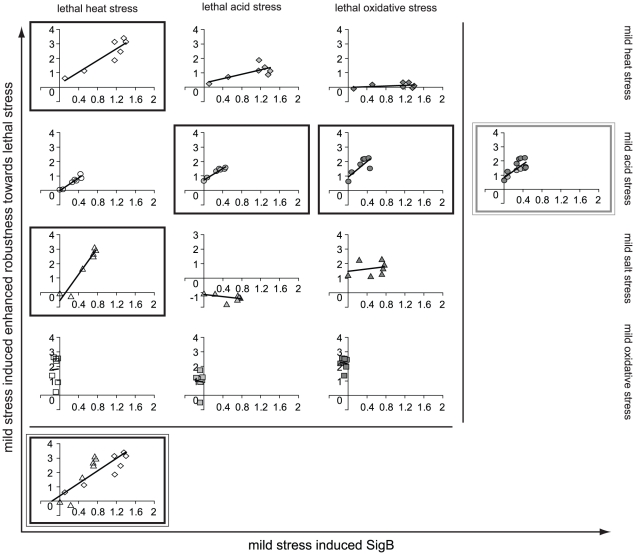
Protein SigB as potential biomarker for mild stress induced enhanced robustness. Induction of protein SigB upon mild stress treatment (43°C, ⋄; pH 5.5, ○; 1.5% NaCl, ▵; 0.1 mM H_2_O_2_, □) was correlated to mild stress induced enhanced robustness towards lethal stress (50°C, open symbols; pH 3.3, light gray filled symbols; 0.2 mM H_2_O_2_, dark gray filled symbols). Each mild stress was applied for 2, 5, 10, 15, 20, 30 and 60 min and induction of SigB and robustness for mild stress pretreated cells (s) was relatively expressed to that of unstressed cells (uns) (log s/uns). Robustness of mild stress pretreated cells and unstressed cells was determined as the number of bacteria surviving the lethal stress treatment compared to the initial number of bacteria. The graph boxes show the conditions for which SigB was qualified as biomarker for mild stress induced enhanced robustness: black box represents a long-term biomarker; double black box represents a convergent-application biomarker; double gray box represents a divergent-application biomarker.

Several biomarkers functioned as long-term or short-term biomarker for mild stress induced enhanced robustness for more than one specific mild stress and lethal stress pair. Moreover, some mild stress conditions did not significantly induce the biomarker and this corresponded to a lack of enhanced robustness following mild stress pretreatment (i.e. the so-called no response conditions, see Figure 6a). To evaluate whether the biomarker could predict the robustness level towards one specific lethal stress originated from multiple mild stress pretreatments, the mild stress induced biomarker and robustness responses were combined and the correlation was tested for significance. When this correlation remained significant (*P*<0.05), then the biomarker was defined as convergent-application biomarker (Figure 6b). The biomarker was defined as divergent-application biomarker when it could predict the robustness level towards multiple lethal stresses upon pretreatment to a specific mild stress (Figure 6c).

### Identification of biomarkers for mild stress induced enhanced robustness

The step-wise procedure was followed to select those conditions for which the candidate-biomarker could predict the enhanced robustness level of the cell following mild stress pretreatment. The SigB protein functioned as a long-term biomarker for mild heat and mild salt stress induced enhanced robustness towards lethal heat stress (Figure 7). SigB was also significantly correlated to lethal heat stress robustness upon mild acid stress pretreatment, but because mild acid stress pretreatment did not significantly induce a robustness advantage towards lethal heat stress (Figure 3), SigB was not identified as a long-term biomarker for this condition. Since SigB could predict the robustness status towards lethal heat stress of both mild heat and mild salt stress pretreated cells, and remained significantly correlated to lethal heat stress robustness when these mild heat and mild salt stress induced responses were combined, SigB was qualified as convergent-application biomarker for lethal heat stress robustness. In addition to SigB protein, also the proteins ClpC and ClpP were identified as convergent-application biomarkers for lethal heat stress robustness, but their predictive potential was related to mild heat and mild acid stress pretreated cells (see Figure S2 and S3 in the supplementary material). Noteworthy, both ClpC and ClpP proteins could predict the enhanced robustness level towards lethal heat stress provided by mild heat pretreatment only for short adaptation intervals (2 to 30 min). Additionally, SigB was suitable to predict the robustness advantage towards both lethal acid and oxidative stress originating from preexposure to mild acid stress. Since SigB could still predict the mild acid stress induced robustness enhancement towards lethal acid and oxidative stress when these responses were combined, it also functioned as divergent-application biomarker.

**Figure 8:**
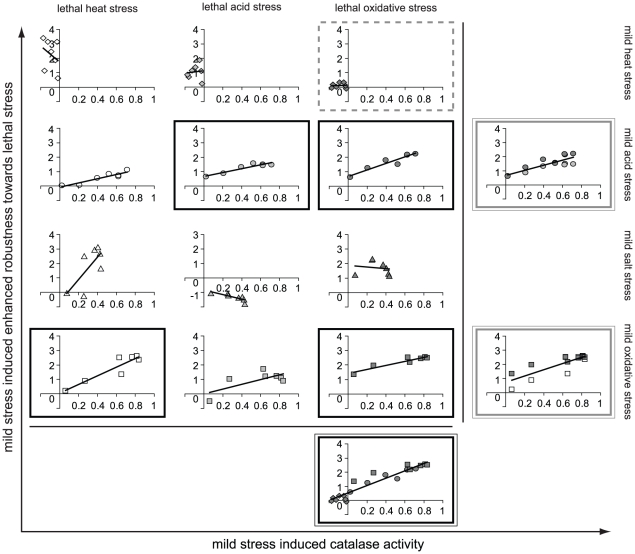
Catalase activity as potential biomarker for mild stress induced enhanced robustness. Induction of catalase activity upon mild stress treatment (43°C, ⋄; pH 5.5, ○; 1.5% NaCl, ▵; 0.1 mM H_2_O_2_, □) was correlated to mild stress induced enhanced robustness towards lethal stress (50°C, open symbols; pH 3.3, light gray filled symbols; 0.2 mM H_2_O_2_, dark gray filled symbols). Each mild stress was applied for 2, 5, 10, 15, 20, 30 and 60 min and induction of catalase activity and robustness for mild stress pretreated cells (s) was relatively expressed to that of unstressed cells (uns) (log s/uns). Robustness of mild stress pretreated cells and unstressed cells was determined as the number of bacteria surviving the lethal stress treatment compared to the initial number of bacteria. The graph boxes show the conditions for which catalase activity was qualified as biomarker for mild stress induced enhanced robustness: black box represents a long-term biomarker; dashed gray box represents a no response condition; double black box represents a convergent-application biomarker; double gray box represents a divergent-application biomarker.

Catalase activity could predict the robustness level towards lethal oxidative stress upon pretreatment to more than two mild stress conditions (Figure 8). Catalase activity functioned as long-term biomarker for enhanced robustness towards lethal oxidative stress originating from mild acid and mild oxidative stress pretreatment. Additionally, mild heat stress did not induce catalase activity and this corresponded to a lack of enhanced robustness towards lethal oxidative stress (no response condition, see Figure 6a). This underlined that the predictive potential of catalase activity was not only restricted to mild stress conditions that induced enhanced robustness, but that it could also predict the lack of enhanced robustness following mild stress pretreatment. Because, catalase activity could still predict the robustness status towards lethal oxidative stress when these three mild stress induced responses were combined, it pointed to the potential of catalase activity to act as a vigorous convergent-application biomarker for lethal oxidative stress robustness. Additionally, catalase activity significantly correlated to robustness towards multiple lethal stresses induced upon pretreatment to mild acid or mild oxidative stress, and functioned therefore also as divergent-application biomarker.

The transcripts of both *clpC* and *clpP* were qualified as convergent-application biomarkers for lethal acid stress robustness upon mild acid and mild salt stress pretreatment (Figure S4 and S5 in the supplementary material). In addition to protein SigB and catalase activity, *clpC* and *clpP* were also suitable to predict the robustness advantage towards multiple lethal stresses, namely lethal acid and oxidative stress, following mild acid stress pretreatment. Furthermore, also the transcript *catE* (Figure S6 in the supplementary material) showed similar divergent predictive potential for those conditions. Detailed supplementary information about the predictive potential of the proteins ClpC and ClpP, and the transcripts *sigB*, *catA*, *catE*, *clpB*, *clpC* and *clpP* is shown in Figures S2, S3, S4, S5, S6, S7, S8, and S9 in the supplementary material, and summarized for all the candidate-biomarkers in Table S3. Noteworthy, the transcript candidate-biomarkers were for various conditions identified as short-term biomarkers rather than long-term biomarkers.

A concluding overview of the predictive potential of the candidate-biomarkers is presented in Table 1 and 2. The SigB protein was suitable to predict the robustness enhancement towards lethal heat stress upon mild heat and mild salt stress pretreatment and was therefore qualified as convergent-application biomarker for lethal heat stress robustness (Table 1, see also Figure 7). Next to SigB, also the proteins ClpC and ClpP were qualified as convergent-application biomarkers for lethal heat stress robustness, but their potential to predict this stress adaptive behavior was related to mild heat and mild acid stress pretreated cells. The predictive potential of the transcripts *clpC* and *clpP* were comparable and both were qualified as convergent-application biomarkers for lethal acid stress robustness following mild acid and mild salt stress preexposure. Catalase activity emerged as a convergent-application biomarker for lethal oxidative stress robustness, which could be elicited by multiple mild stress pretreatments (e.g. mild heat, acid and oxidative stress) (see also Figure 8). Several biomarkers were correlated to robustness towards multiple lethal stresses upon pretreatment to a specific mild stress (Table 2). In addition to SigB, four other biomarkers − *catE*, *clpC*, *clpP* and catalase activity − showed similar predictive potential by emerging as divergent-application biomarker for lethal acid and lethal oxidative stress robustness following mild acid stress pretreatment. Therefore, this study also showed that various biomarkers can have similar predictive potential.

**Table 1:** Convergent-application biomarkers for mild stress induced enhanced robustness.

Convergent-application biomarker multiple mild stresses, lethal stress specific	Mild stress^a^	Lethal stress
SigB	heat, salt	heat
ClpC	heat, *acid*	heat
ClpP	heat, *acid*	heat
*clpC*	acid, *salt*	acid
*clpP*	acid, *salt*	acid
catalase activity	*heat*, acid, H_2_O_2_	H_2_O_2_

a italic text indicates that mild stress treatment did not significantly induce the biomarker and also did not provide a robustness enhancement towards lethal stress upon pretreatment (no response condition).

**Table 2:** Divergent-application biomarkers for mild stress induced enhanced robustness.

Divergent-application biomarker mild stress specific, multiple lethal stresses	Mild stress	Lethal stress
SigB	acid	acid, H_2_O_2_
*catE*	acid	acid, H_2_O_2_
*clpC*	acid	acid, H_2_O_2_
*clpP*	acid	acid, H_2_O_2_
catalase activity	acid	acid, H_2_O_2_
catalase activity	H_2_O_2_	heat, H_2_O_2_

## Discussion

The adaptive stress response of bacteria is a crucial mode of cellular protection and allows bacteria to survive in changing environments. Cellular biomarkers that are quantitatively correlated to stress adaptive behavior will allow to predict the impact of changing environments on bacterial fitness, robustness and survival. The availability of complete genome sequences of a wide variety of bacteria has been instrumental in the development of functional genomics technologies that integrate molecular biology and classical physiology to further understand bacterial stress adaptation mechanisms [27]. These technologies, that make use of holistic and unbiased approaches, can direct our search for crucial cellular components that may function as biomarkers for stress adaptive behavior and subsequent enhanced robustness under challenging conditions. In this study, we identified various transcript biomarkers and also biomarkers at protein and enzyme activity levels, all with predictive quality. We proposed a framework for selecting those cellular biomarkers for mild stress induced enhanced robustness and we systematically evaluated the predictive potential of the candidate-biomarkers for our model-organism *B. cereus*. Based on a genome-wide comparison of transcriptome profiles of *B. cereus* cells exposed to four mild stress conditions, several potential candidate-biomarkers were selected. These were the general stress response transcriptional regulator σ^B^, catalases involved in H_2_O_2_-scavenging, and chaperones and ATP-dependent Clp proteases involved in protein repair and maintenance. Since several mild stress conditions induced the transcription of genes involved in these stress responses, these findings support their significance in *B. cereus* general stress response. Activation of cellular mechanisms involved in stress response-signaling and -regulation upon exposure to stress conditions as well as activation of systems involved in oxidative stress defense and maintenance of protein quality might be an essential and general mode of microbial stress adaptation and enhancement of bacterial robustness. Therefore, the canonical induction of these stress responses may extend beyond the species *B. cereus*, and has been demonstrated for other bacteria including *Bacillus subtilis*, *Listeria monocytogenes* and *Escherichia coli* upon exposure to stress conditions [19], [28], [29], but also in yeast by a wide variety of environmental changes [16], [17]. In the present study, the induction of σ^B^, catalases, chaperones and ATP-dependent Clp proteases were quantitatively determined at transcript, protein and/or activity level upon mild stress treatment and these responses were correlated to mild stress induced enhanced robustness towards lethal stress, aiming to evaluate their predictive biomarker potential at these different functional cell levels. Our study shows that the predictive potential of cellular indicators is highly influenced by the functional cell level at which the indicator is measured. Catalase activity and the SigB protein were identified as long-term biomarkers for various adaptive stress responses. Moreover, the lack of induction of catalase activity in mild heat stress adapted cells corresponded to the lack of enhanced robustness towards lethal oxidative stress, and therefore, it also showed to have predictive potential for this condition where mild stress pretreatment did not provide cross-protection. Both catalase activity and the SigB protein could be employed as convergent- and divergent-application biomarkers as their predictive ability was not restricted to a single mild stress and lethal stress pair. These findings underlined the high predictive quality of these biomarkers and pointed to a promising role in prediction of stress adaptive behavior. In contrast, the *catA* transcript of the main vegetative catalase was not suitable to predict the robustness status towards lethal stress of mild stress pretreated cells for any of the tested conditions, underpinning the significance to evaluate the predictive potential of cellular indicators at different functional cell levels. The *sigB* transcript acted as short-term biomarker and predicted the robustness enhancement towards lethal heat stress provided by mild heat stress preexposure for rather short mild heat stress adaptation intervals (up to 15 min). This demonstrated that the predictive potential of biomarkers was manifested across multiple time scales. The better predictive potential of enzyme activity and proteins compared to transcripts might be a reflection of the transient nature of the mRNA levels of some of the selected candidate-biomarkers.

The adaptive traits of bacteria can antagonize food processing strategies that rely on combining mild preservation treatments to control bacterial growth of spoilage and pathogenic bacteria, but are also crucial for various industrial functional food applications. The identification of potential biomarkers for mild stress induced enhanced robustness towards lethal stresses contributes to a better understanding of bacterial stress adaptation mechanisms and can guide our search to control and/or exploit these adaptive traits. The level of robustness enhancement provided by mild stress preexposure was demonstrated to depend on the type and concentration of mild stress applied and the selected lethal stress condition. These different ranges of enhanced (cross-)protection were not taken into consideration in this study when the predictive quality of the candidate-biomarkers was evaluated. The focus of the present study was to design a framework for identification of biomarkers that were able to predict the robustness status of mild stress adapted cells. This can be the basis for further characterizing predictive potential of promising biomarkers by addressing the robustness enhancement ranges provided by mild stress pretreatment. Promising candidate-biomarkers might be species- and genus-specific because the functional conservation of stress-related cellular factors differs among species and genera, and the predictive quality of biomarkers might even be strain specific. The role and regulation of key regulators of general stress responses as well as cellular mechanisms that are crucial for controlling protein quality and defending against oxidative stresses have been shown to differ between closely-related genera. The transcriptional regulator σ^B^ functions as central regulator of general stress responses in Gram-positive bacteria with a low GC content including the genera *Bacillus*, *Staphylococcus* and *Listeria* with variations in regulon-members and -size [19], [23], [24], [30]–[35]. This sigma factor is absent in various lactic acid bacteria [10], [36], [37] suggesting that lactic acid bacteria have developed different stress regulatory networks. Clp proteases and chaperones and the main vegetative catalase, predominantly controlled by the CtsR and/or HrcA repressors [38], [39] and by PerR [40]–[43] in low GC Gram-positive bacteria, respectively, are widely conserved in microorganisms [39], [44], [45] and play indispensable roles in cellular repair and defense strategies. Despite apparent variations in their mechanisms of expression control between species and genera, these canonical stress-related components are among the most consistently induced components in microbial stress responses and ubiquitous in biology. We showed that these stress indicators can serve as predictors of stress adaptive behavior in *B. cereus* and it is conceivable that they may also be employed in other microorganisms. Variations in functional conservations and regulatory circuits necessitates a profound validation of biomarker quality, and our study provides a quantitative approach to systematically search for these biomarkers and to evaluate their predictive potential.

In conclusion, we presented a framework for identifying cellular biomarkers for stress adaptive behavior and to evaluate their predictive potential at transcript, protein and activity level. This quantitative approach opens avenues towards prediction of microbial performance using cellular biomarkers which can serve to early detect and control adaptive behavior that results in enhanced robustness.

## Materials and Methods

### Bacterial strain and preculturing conditions

*Bacillus cereus* ATCC 14579 was used as model-organism throughout the study. Stock cultures grown in brain heart infusion (BHI, Becton Dickinson, France) broth were stored at −80°C in 25% (v/v) glycerol. To prepare precultures, 10 ml BHI broth was inoculated with a droplet of the stock culture and incubated overnight at 30°C with shaking at 200 rpm.

### Mild stress treatment

The precultures were inoculated in Erlenmeyer flasks containing 50 ml fresh BHI broth and incubated at 30°C with shaking at 200 rpm until the cells were exponentially growing (absorbance value at 600 nm of 0.4 to 0.5; unstressed, reference condition). Upon reaching this optical density, the exponentially growing cells were treated with four different mild stresses for 2, 5, 10, 15, 20, 30 and 60 min with shaking at 200 rpm. The following mild stress conditions were applied: heat stress (43°C); acid-shock (pH 5.5, adjusted with 37% hydrochloric acid, at 30°C); osmotic-upshift (1.5% [w/v] sodium chloride, at 30°C); and oxidative stress (0.1 mM H_2_O_2_, at 30°C). Preliminary experiments had demonstrated that preexposure for 15 min to those selected conditions resulted in optimal heat resistance (Figure S1 in the supplementary material).

### Determination of lethal stress robustness following mild stress pretreatment

Both unstressed and mild stress treated cells were subsequently exposed to three lethal stress conditions to determine their specific robustness using the inactivation procedure described previously [21]. The following inactivation conditions were chosen: heat stress (50°C); low pH (pH 3.3, adjusted with 37% hydrochloric acid, at 30°C); and oxidative stress (0.2 mM H_2_O_2_, at 30°C). Before and after inactivation treatment, samples were taken and decimal dilutions were made in peptone saline solution (1 g neutralized bacteriological peptone [Oxoid, United Kingdom] supplemented with 8.5 g sodium chloride per liter). After acid-inactivation treatment, samples were decimally diluted in BHI broth to ensure no further acid-inactivation during diluting. The appropriate dilutions were surface plated, in duplicate, on BHI agar plates (BHI broth supplemented with 15 g agar [Oxoid, United Kingdom] per liter) using a spiral plater (Eddy Jet; IUL Instruments, Spain) and the plates were incubated at 30°C for 16 to 24 h. The experiments to inactivate unstressed and mild stress pretreated cells were reproduced 2 to 3 times on different days. The robustness of both unstressed and mild stress pretreated cells was determined as the number of microorganism surviving the inactivation treatment, *N(t)*, compared to the initial number of microorganism before the inactivation treatment at *t* = 0, *N(0)*. *t*-Tests were performed to compare the log_10_-robustness of mild stress pretreated cells to that of unstressed cells (with *P*<0.05 as significance threshold).

### Determination of candidate-biomarker induction upon mild stress treatment

Ten candidate-biomarkers were quantitatively measured before and after mild stress treatment for 2 to 60 min, namely, catalase activity, the proteins SigB, ClpC and ClpP, and the transcripts *sigB*, *catA*, *catE*, *clpB*, *clpC*, *clpP*. The experimental procedures followed to measure and to quantify the responses of these candidate-biomarkers upon mild stress treatment are described below.

#### Catalase activity assay

A previously described procedure was used to determine the catalase activity of unstressed cells and mild stress treated cells [21], [26]. Briefly, cells were washed in phosphate-buffered saline and subsequently exposed to hydrogen peroxide, and the decrease in absorbance at 240 nm was measured over time at 30°C with a spectrophotometer (Spectramax Plus 384; Molecular Devices, USA). One unit of catalase activity was defined as a decrease in absorbance at 240 nm of 1 unit per minute. The rate of decrease for each sample was corrected for the amount of cells used in the assay and standardized to absorbance value of 0.5 at 600 nm. For all mild stress treatment intervals, three biologically independent catalase activity experiments were performed.

The catalase activity was also visualized by catalase activity staining on a native polyacrylamide gel. For that, total proteins were extracted from unstressed and mild stress treated cultures following a similar procedure as previously described [15], [26]. Subsequently, fifty micrograms of protein extracts were separated on a native 10% Tris-HCl polyacrylamide gel (Criterion; Bio-Rad Laboratories, USA). Catalase activity was visualized as described previously [46], which results in yellow catalase bands against a dark-green background.

#### Western blotting

Total cellular protein was extracted from four biologically independent cultures of unstressed and mild stress treated cells. Forty micrograms of protein extracts were separated by using 15% Tris-HCl gels for SigB and ClpP, and 7.5% Tris-HCl gels for ClpC. Immunoblotting was performed as described previously [47] with anti-SigB antibodies raised against the SigB protein of *B. cereus* [47], and anti-ClpC and anti-ClpP antibodies raised against these proteins of *B. subtilis* [15]. Immunocomplexes were visualized using Chemiluminescent Peroxidase Substrate-3 (Sigma-Aldrich, Germany) and scanned with a Chemiluminescence scanner with ChemiDoc XRS software (Bio-Rad Laboratories, USA). The band intensity was quantified using Quantity One software (version 4.6.1; Bio-Rad Laboratories, USA) with background subtraction.

#### RNA isolation and RT-PCR

RNA was isolated from two biologically independent cultures of unstressed and mild stress treated cells. For that, 10 ml of culture was transferred into a 50-ml Falcon tube, and spun down at 13,000×*g* for 30 s at 4°C. After the supernatant was decanted, the cell pellets were immediately resuspended in 1 ml of TRI-reagent (Ambion, UK) and snap-frozen in liquid nitrogen. The RNA was further extracted as described previously [47]. Synthesis of cDNA and real time(RT)-PCR was carried out as described previously [48], with *16S-rRNA* as reference gene, and *sigB*, *catA*, *catE*, *clpB*, *clpC* and *clpP* as target genes. The primers of the reference and target genes are listed in Table S4 in the supplementary material. The relative expression ratios of the target genes were calculated as previously described [49].

#### Evaluation of candidate-biomarker induction

The relative induction levels of the candidate-biomarkers in mild stress treated cells compared to unstressed cells were log_10_-transformed and averaged for the biologically independent reproductions for each mild stress treatment time point. *t*-Tests were performed to evaluate whether the induction of the candidate-biomarker in mild stress treated cells was statistically significant (with *P*<0.05 as significance threshold).

### Correlation between mild stress induced robustness and candidate-biomarkers responses

The robustness of mild stress pretreated cells (s) was also relatively expressed to that of unstressed cultures (uns) and subsequently log_10_-transformed, 
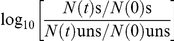
, and averaged for the biological reproductions. Then, for each candidate-biomarker, the mild stress induced candidate-biomarker responses were quantitatively correlated to mild stress induced robustness towards lethal stress per mild stress and lethal stress pair. The Pearson correlation coefficient *r* was calculated to test the significance of each correlation using PASW software (version 17.0.3).

## Supporting Information

Figure S1 Selection of mild stress condition for mild stress adaptation experiments. Inactivation kinetics of *Bacillus cereus* ATCC 14579 cells at 50°C following mild stress pretreatment for 15 min. Four mild stress conditions were used for pretreatment, namely, heat stress (a), acid-shock (b), osmotic-upshift (c) and oxidative stress (d), respectively. Pretreatment with 43°C (a), pH 5.5 (b), 1.5% NaCl (c) and 0.1 mM H_2_O_2_ (d) resulted in subsequent optimal heat resistance.
(1.00 MB EPS)

Figure S2 Protein ClpC as potential biomarker for mild stress induced enhanced robustness. Induction of protein ClpC upon mild stress treatment (43°C, pH 5.5, 1.5% NaCl, 0.1 mM H_2_O_2_) was correlated to mild stress induced enhanced robustness towards lethal stress (50°C, open symbols; pH 3.3, light gray filled symbols; 0.2 mM H_2_O_2_, dark gray filled symbols). Each mild stress was applied for 2, 5, 10, 15, 20, 30 and 60 min and induction of ClpC and robustness for mild stress pretreated cells (s) was relatively expressed to that of unstressed cells (uns) (log s/uns). Robustness of mild stress pretreated cells and unstressed cells was determined as the number of bacteria surviving the lethal stress treatment compared to the initial number of bacteria. The graph boxes show the conditions for which ClpC was qualified as biomarker for mild stress induced enhanced robustness: gray box represents a short-term biomarker; dashed gray box represents a no response condition; double black box represents a convergent-application biomarker. Short-term biomarkers are only correlated to robustness for short-term mild stress adaptation intervals, excluding long-term intervals (black filled symbol).
(1.10 MB EPS)

Figure S3 Protein ClpP as potential biomarker for mild stress induced enhanced robustness. Induction of protein ClpP upon mild stress treatment (43°C, pH 5.5, 1.5% NaCl, 0.1 mM H_2_O_2_) was correlated to mild stress induced enhanced robustness towards lethal stress (50°C, open symbols; pH 3.3, light gray filled symbols; 0.2 mM H_2_O_2_, dark gray filled symbols). Each mild stress was applied for 2, 5, 10, 15, 20, 30 and 60 min and induction of ClpP and robustness for mild stress pretreated cells (s) was relatively expressed to that of unstressed cells (uns) (log s/uns). Robustness of mild stress pretreated cells and unstressed cells was determined as the number of bacteria surviving the lethal stress treatment compared to the initial number of bacteria. The graph boxes show the conditions for which ClpP was qualified as biomarker for mild stress induced enhanced robustness: gray box represents a short-term biomarker; dashed gray box represents a no response condition; double black box represents a convergent-application biomarker. Short-term biomarkers are only correlated to robustness for short-term mild stress adaptation intervals, excluding long-term intervals (black filled symbol).
(1.10 MB EPS)

Figure S4 Transcript *clpC* as potential biomarker for mild stress induced enhanced robustness. Induction of transcript *clpC* upon mild stress treatment (43°C, pH 5.5, 1.5% NaCl, 0.1 mM H_2_O_2_) was correlated to mild stress induced enhanced robustness towards lethal stress (50°C, open symbols; pH 3.3, light gray filled symbols; 0.2 mM H_2_O_2_, dark gray filled symbols). Each mild stress was applied for 2, 5, 10, 15, 20, 30 and 60 min and induction of *clpC* and robustness for mild stress pretreated cells (s) was relatively expressed to that of unstressed cells (uns) (log s/uns). Robustness of mild stress pretreated cells and unstressed cells was determined as the number of bacteria surviving the lethal stress treatment compared to the initial number of bacteria. The graph boxes show the conditions for which *clpC* was qualified as biomarker for mild stress induced enhanced robustness: black box represents a long-term biomarker; gray box represents a short-term biomarker; dashed gray box represents a no response condition; double black box represents a convergent-application biomarker; double gray box represents a divergent-application biomarker. Short-term biomarkers are only correlated to robustness for short-term mild stress adaptation intervals, excluding long-term intervals (black filled symbol).
(1.12 MB EPS)

Figure S5 Transcript *clpP* as potential biomarker for mild stress induced enhanced robustness. Induction of transcript *clpP* upon mild stress treatment (43°C, pH 5.5, 1.5% NaCl, 0.1 mM H_2_O_2_) was correlated to mild stress induced enhanced robustness towards lethal stress (50°C, open symbols; pH 3.3, light gray filled symbols; 0.2 mM H_2_O_2_, dark gray filled symbols). Each mild stress was applied for 2, 5, 10, 15, 20, 30 and 60 min and induction of *clpP* and robustness for mild stress pretreated cells (s) was relatively expressed to that of unstressed cells (uns) (log s/uns). Robustness of mild stress pretreated cells and unstressed cells was determined as the number of bacteria surviving the lethal stress treatment compared to the initial number of bacteria. The graph boxes show the conditions for which *clpP* was qualified as biomarker for mild stress induced enhanced robustness: black box represents a long-term biomarker; gray box represents a short-term biomarker; dashed gray box represents a no response condition; double black box represents a convergent-application biomarker; double gray box represents a divergent-application biomarker. Short-term biomarkers are only correlated to robustness for short-term mild stress adaptation intervals, excluding long-term intervals (black filled symbol).
(1.12 MB EPS)

Figure S6 Transcript *catE* as potential biomarker for mild stress induced enhanced robustness. Induction of transcript *catE* upon mild stress treatment (43°C, pH 5.5, 1.5% NaCl, 0.1 mM H_2_O_2_) was correlated to mild stress induced enhanced robustness towards lethal stress (50°C, open symbols; pH 3.3, light gray filled symbols; 0.2 mM H_2_O_2_, dark gray filled symbols). Each mild stress was applied for 2, 5, 10, 15, 20, 30 and 60 min and induction of *catE* and robustness for mild stress pretreated cells (s) was relatively expressed to that of unstressed cells (uns) (log s/uns). Robustness of mild stress pretreated cells and unstressed cells was determined as the number of bacteria surviving the lethal stress treatment compared to the initial number of bacteria. The graph boxes show the conditions for which *catE* was qualified as biomarker for mild stress induced enhanced robustness: black box represents a long-term biomarker; gray box represents a short-term biomarker; double gray box represents a divergent-application biomarker. Short-term biomarkers are only correlated to robustness for short-term mild stress adaptation intervals, excluding long-term intervals (black filled symbols).
(0.98 MB EPS)

Figure S7 Transcript *sigB* as potential biomarker for mild stress induced enhanced robustness. Induction of transcript *sigB* upon mild stress treatment (43°C, pH 5.5, 1.5% NaCl, 0.1 mM H_2_O_2_) was correlated to mild stress induced enhanced robustness towards lethal stress (50°C, open symbols; pH 3.3, light gray filled symbols; 0.2 mM H_2_O_2_, dark gray filled symbols). Each mild stress was applied for 2, 5, 10, 15, 20, 30 and 60 min and induction of *sigB* and robustness for mild stress pretreated cells (s) was relatively expressed to that of unstressed cells (uns) (log s/uns). Robustness of mild stress pretreated cells and unstressed cells was determined as the number of bacteria surviving the lethal stress treatment compared to the initial number of bacteria. The graph boxes show the conditions for which *sigB* was qualified as biomarker for mild stress induced enhanced robustness: black box represents a long-term biomarker; gray box represents a short-term biomarker. Short-term biomarkers are only correlated to robustness for short-term mild stress adaptation intervals, excluding long-term intervals (black filled symbols).
(1.07 MB EPS)

Figure S8 Transcript *catA* as potential biomarker for mild stress induced enhanced robustness. Induction of transcript *catA* upon mild stress treatment (43°C, pH 5.5, 1.5% NaCl, 0.1 mM H_2_O_2_) was correlated to mild stress induced enhanced robustness towards lethal stress (50°C, open symbols; pH 3.3, light gray filled symbols; 0.2 mM H_2_O_2_, dark gray filled symbols). Each mild stress was applied for 2, 5, 10, 15, 20, 30 and 60 min and induction of *catA* and robustness for mild stress pretreated cells (s) was relatively expressed to that of unstressed cells (uns) (log s/uns). Robustness of mild stress pretreated cells and unstressed cells was determined as the number of bacteria surviving the lethal stress treatment compared to the initial number of bacteria. For non of the mild stress and lethal stress pairs, *catA* was qualified as biomarker.
(1.06 MB EPS)

Figure S9 Transcript *clpB* as potential biomarker for mild stress induced enhanced robustness. Induction of transcript *clpB* upon mild stress treatment (43°C, pH 5.5, 1.5% NaCl, 0.1 mM H_2_O_2_) was correlated to mild stress induced enhanced robustness towards lethal stress (50°C, open symbols; pH 3.3, light gray filled symbols; 0.2 mM H_2_O_2_, dark gray filled symbols). Each mild stress was applied for 2, 5, 10, 15, 20, 30 and 60 min and induction of *clpB* and robustness for mild stress pretreated cells (s) was relatively expressed to that of unstressed cells (uns) (log s/uns). Robustness of mild stress pretreated cells and unstressed cells was determined as the number of bacteria surviving the lethal stress treatment compared to the initial number of bacteria. The graph boxes show the conditions for which *clpB* was qualified as biomarker for mild stress induced enhanced robustness: black box represents a long-term biomarker; dashed gray box represents a no response condition.
(1.06 MB EPS)

Table S1 Overlap of transcriptome responses upon mild stress treatment
(0.07 MB DOC)

Table S2 Mild stress induced (cross-)protection towards lethal stress
(0.03 MB DOC)

Table S3 Biomarkers for mild stress induced enhanced robustness towards lethal stress
(0.06 MB DOC)

Table S4 RT-PCR primers used in this study
(0.03 MB DOC)
