# Supplementary material for: Short- and Long-Term Biomarkers for Bacterial Robustness: A Framework for Quantifying Correlations between Cellular Indicators and Adaptive Behavior
Source: PLoS One. 2010 Oct 29;5(10):e13746. doi: 10.1371/journal.pone.0013746 (PMC2966415; doi:10.1371/journal.pone.0013746)
Supplement: Table S1 — Overlap of transcriptome responses upon mild stress treatment (0.07 MB DOC) [file pone.0013746.s010.doc]

**Table S1. Overlap of transcriptome responses upon mild** stress treatment

|  |  | **Mild stress condition^a^** | | | |
| --- | --- | --- | --- | --- | --- |
| **Gene-no^b, c, d^** | **Annotation** | **Heat** | **Acid** | **Salt** | **H_2_O_2_** |
| BC0099^d^ | CtsR transcriptional regulator | 5.54 | 5.07 | 2.08 | 2.02 |
| BC0100^d^ | ClpC ATPase | 11.53 | 6.66 | 2.64 | 2.16 |
| BC0101^d^ | Arginine kinase | 12.66 | 6.69 | 2.84 | 2.29 |
| BC0102^d^ | ClpC | 12.00 | 5.83 | 2.66 | 2.01 |
| BC0304 | FrnE protein | 2.04 | 2.70 | 1.29 | 3.69 |
| BC0377^c^ | Alkyl hydroperoxide reductase | 1.32 | 5.07 | 3.22 | 9.76 |
| BC0387 | hypothetical protein | 1.34 | 1.68 | 1.51 | 1.61 |
| BC0503 | hypothetical protein | 2.30 | 2.47 | 1.73 | 1.66 |
| BC0584 | Acetyltransferase | 2.15 | 1.58 | 1.80 | 2.06 |
| BC0613 | ArsR transcriptional regulator | 2.31 | 2.99 | 3.08 | 1.76 |
| BC0993 | PadR transcriptional regulator | 1.97 | 1.53 | 1.37 | 1.54 |
| BC1003^b^ | Anti-sigmaB factor | 52.41 | 16.25 | 12.96 | 1.63 |
| BC1005^b^ | Bacterioferritin | 56.05 | 29.56 | 63.31 | 1.63 |
| BC1006^b^ | PP2C phosphatase | 5.13 | 3.37 | 6.18 | 2.89 |
| BC1154^b^ | Ferrochelatase | 3.40 | 12.23 | 10.87 | 6.73 |
| BC1155^b, c^ | Catalase CatA | 2.23 | 11.83 | 8.10 | 17.77 |
| BC1246 | NADH dehydrogenase | 2.12 | 2.00 | 1.34 | 2.94 |
| BC1786 | Multidrug resistance protein B | 1.65 | 4.61 | 1.95 | 6.54 |
| BC1803 | Manganese transport protein | 2.14 | 4.67 | 1.75 | 2.36 |
| BC2241 | Succinate-semialdehyde dehydrogenase | 1.90 | 4.56 | 1.81 | 2.46 |
| BC3402 | Arsenate reductase family protein | 1.90 | 2.52 | 3.04 | 2.80 |
| BC3647 | N-ethylmaleimide reductase | 2.24 | 2.05 | 1.34 | 1.97 |
| BC3668 | LysR transcriptional regulator | 3.20 | 2.08 | 1.99 | 2.16 |
| BC4211 | LacI transcriptional regulator | 1.66 | 3.08 | 1.83 | 2.09 |
| BC4313 | GrpE protein | 2.80 | 1.60 | 1.82 | 2.25 |
| BC4314^d^ | HrcA transcription repressor | 5.01 | 2.41 | 2.41 | 2.31 |
| BC4775 | Phosphoglycerol transferase | 2.02 | 1.82 | 1.75 | 2.22 |
| BC4866 | Glucose-1-phosphate adenylyltransferase | 1.42 | 2.06 | 1.91 | 1.80 |
| BC5401 | Lipase/acylhydrolase | 2.24 | 2.40 | 1.28 | 2.75 |

^a^The comparison of the genome-wide transcriptome profiles of *B. cereus* ATCC 14579 upon treatment to four mild stress conditions − heat stress [23], acid-shock [22], osmotic-upshift [21, M. Mols and A. Streng, unpublished data] and oxidative stress [20] − for 10 min, revealed a limited number of genes that were differentially expressed upon treatment to all four mild stress conditions. The values denote the transcription ratios of these genes for mild stress treated cells compared to unstressed cells (mid-exponential growth phase with OD_600 nm_ 0.4 to 0.5). The microarray data of the mild heat, acid, salt and oxidative stress adaptation experiments have been deposited in the GEO database with accession numbers GSE6005, GSE15140, GSE13713 and GSE18807, respectively. The genes of which the expression ratios were at least five upon treatment to one mild stress condition and at least two upon treatment to two other mild stress conditions, represented three functional categories: members of the general stress regulon controlled by the transcriptional regulator σ^B^ [23,24]; cellular defense mechanisms against oxidative stress; repair and maintenance of cellular protein quality.

^b^Gene is part of the σ^B^-regulon.

^c^Gene is associated with cellular defense mechanisms against oxidative stress.

^d^Gene is associated with repair and maintenance of cellular protein quality.
