# Supplementary material for: Short- and Long-Term Biomarkers for Bacterial Robustness: A Framework for Quantifying Correlations between Cellular Indicators and Adaptive Behavior
Source: PLoS One. 2010 Oct 29;5(10):e13746. doi: 10.1371/journal.pone.0013746 (PMC2966415; doi:10.1371/journal.pone.0013746)
Supplement: Table S2 — Mild stress induced (cross-)protection towards lethal stress (0.03 MB DOC) [file pone.0013746.s011.doc]

**Table S2. Mild stress induced (cross-)protection towards lethal stress**

|  | **Lethal stress^a^** | | |
| --- | --- | --- | --- |
| **Mild stress** | **Heat** | **Acid** | **H_2_O_2_** |
| Heat | + | + |  |
| Acid |  | + | + |
| Salt | + |  | + |
| H_2_O_2_ | + | + | + |

^a^Plus symbol (+) represents enhanced robustness towards lethal stress (heat, 50°C; acid, pH 3.3; oxidative stress, 0.2 mM H_2_O_2_) upon mild stress pretreatment (heat, 43°C; acid, pH 5.5; salt, 1.5% NaCl; oxidative stress, 0.1 mM H_2_O_2_).
