# Supplementary material for: Short- and Long-Term Biomarkers for Bacterial Robustness: A Framework for Quantifying Correlations between Cellular Indicators and Adaptive Behavior
Source: PLoS One. 2010 Oct 29;5(10):e13746. doi: 10.1371/journal.pone.0013746 (PMC2966415; doi:10.1371/journal.pone.0013746)
Supplement: Table S3 — Biomarkers for mild stress induced enhanced robustness towards lethal stress (0.06 MB DOC) [file pone.0013746.s012.doc]

**Table S3. Biomarkers for mild stress induced enhanced robustness towards lethal stress**

|  |  | **Mild stress^a^** | | | |
| --- | --- | --- | --- | --- | --- |
| **Biomarker** | **Lethal stress** | **Heat** | **Acid** | **Salt** | **H_2_O_2_** |
| SigB | Heat | L |  | L |  |
|  | Acid |  | L |  |  |
|  | H_2_O_2_ |  | L |  |  |
| ClpC | Heat | S | N |  |  |
|  | Acid |  |  | N |  |
|  | H_2_O_2_ |  |  |  |  |
| ClpP | Heat | S | N |  |  |
|  | Acid |  |  | N |  |
|  | H_2_O_2_ |  |  |  |  |
| Catalase activity | Heat |  |  |  | L |
|  | Acid |  | L |  |  |
|  | H_2_O_2_ | N | L |  | L |
| *sigB* | Heat | S |  |  |  |
|  | Acid |  |  |  |  |
|  | H_2_O_2_ |  |  | L |  |
| *catA* | Heat |  |  |  |  |
|  | Acid |  |  |  |  |
|  | H_2_O_2_ |  |  |  |  |
| *catE* | Heat | S |  |  |  |
|  | Acid |  | S |  |  |
|  | H_2_O_2_ |  | L | L |  |
| *clpB* | Heat |  |  |  |  |
|  | Acid |  |  | N |  |
|  | H_2_O_2_ |  | L |  |  |
| *clpC* | Heat |  |  |  |  |
|  | Acid |  | S | N |  |
|  | H_2_O_2_ |  | L |  |  |
| *clpP* | Heat |  |  |  |  |
|  | Acid |  | S | N |  |
|  | H_2_O_2_ |  | L |  |  |

^a^Long-term biomarker, L; Short-term biomarker, S; No response condition, N.
