# Supplementary material for: Short- and Long-Term Biomarkers for Bacterial Robustness: A Framework for Quantifying Correlations between Cellular Indicators and Adaptive Behavior
Source: PLoS One. 2010 Oct 29;5(10):e13746. doi: 10.1371/journal.pone.0013746 (PMC2966415; doi:10.1371/journal.pone.0013746)
Supplement: Table S4 — RT-PCR primers used in this study (0.03 MB DOC) [file pone.0013746.s013.doc]

**Table S4. RT-PCR primers used in this study**

| **Primername** | **Gene-no** | **Sequence (5’-3’)** |
| --- | --- | --- |
| 16SrRNA_Forw | BC0007 | AATTCGAAGCAACGCGAAGA |
| 16SrRNA_Rev | BC0007 | ACACGAGCTGACGACAACCA |
| sigB_Forw | BC1004 | CAATGTGATGAAGCGCAGGA |
| sigB_Rev | BC1004 | CGGTCCGCCTTTTGAATAGC |
| catA_Forw | BC1155 | CTGGAAACCACAGCAAGGTG |
| catA_Rev | BC1155 | CGAACAGGTCACGAGTAGCA |
| catE_Forw | BC0863 | CGACTCATTAACACAGCAGGAA |
| catE_Rev | BC0863 | CTTTGAGCTTCATCCCATACTA |
| clpB_Forw | BC1168 | TTTCACGCTGGACTGGTATTC |
| clpB_Rev | BC1168 | TCCGATGACACGCTCTGATAA |
| clpC_Forw | BC0102 | TAATCCGTGAAGGTGAAGGTGT |
| clpC_Rev | BC0102 | CACTTGTTGTCTTGCTTTGTTG |
| clpP_Forw | BC5152 | TGGTGGAGCACAAGGTCAAG |
| clpP_Rev | BC5152 | GCGGTCAGCAAGAATTTGG |
